# Effects of oral health interventions on cognition of people with dementia: a systematic review with meta-analysis

**DOI:** 10.1186/s12903-024-04750-4

**Published:** 2024-09-03

**Authors:** Haiying Guo, Zongqin Wang, Chun Hung Chu, Alice Kit Ying Chan, Edward Chin Man Lo, Chloe Meng Jiang

**Affiliations:** 1 State Key Laboratory of Oral & Maxillofacial Reconstruction and Regeneration, Key Laboratory of Oral Biomedicine Ministry of Education, Hubei Key Laboratory of Stomatology, School & Hospital of Stomatology, Wuhan University, Wuhan, China; 2 Faculty of Dentistry, The University of Hong Kong, Hong Kong, China; 3 Wuhan Mental Health Center, Wuhan, China

**Keywords:** Oral health, Oral hygiene practice, Oral exercise, Cognition, Dementia, Older adults

## Abstract

**Background:**

Increasing studies have shown that poor oral health contributes to the progression of dementia. It is meaningful to find out the role of oral health interventions in maintaining people’s cognition levels and delaying the progression of dementia. Thus, we conducted this review to summarize the present evidence on the effect of oral health interventions on the cognition change of people with dementia.

**Methods:**

Literature search was conducted in the databases of PubMed, Embase, Web of Science, Cochrane library, and Dentistry and Oral Sciences by two independent reviewers from inception to 6 March 2024. Clinical studies such as randomized controlled trials reporting on the effect of oral health interventions on the cognition of people with dementia were included in this review. Mini-Mental State Examination (MMSE) scores were used to measure cognition level. The mean deviation (MD), generated by subtracting the baseline MMSE score from the MMSE score at follow-up was used to assess the change in cognition. Studies with oral hygiene practice as an oral health intervention were further conducted with a meta-analysis.

**Results:**

A total of 6646 references were identified by the literature search, and 5 studies were eligible to be included in this review. Among the included studies, 4 studies reported the cognition change after having various oral hygiene practice as oral health intervention, while the other study adopted oral exercises as the intervention. Two studies presented positive MD values after intervention provided, indicating improved cognition level at follow-up (MD = 0.6, MD = 0.9, respectively). Another two studies reported less cognition deterioration with smaller absolute MD values in the intervention group, (intervention vs. control, -0.18 vs. -0.75, *p* < 0.05 and − 1.50 vs. -3.00, *p* < 0.05, respectively). The random-effect model was selected in the meta-analysis, and the weighted mean difference (WMD) was 1.08 (95% confidence interval, 0.44 to 1.71), favoring the intervention group.

**Conclusion:**

With limited evidence, oral hygiene care may play a positive role in maintaining the cognition level of people with dementia. However, further studies are needed to provide direct evidence on the effectiveness of oral health interventions on oral health conditions as well as cognition status and to disclose the rationale behind it.

**Supplementary Information:**

The online version contains supplementary material available at 10.1186/s12903-024-04750-4.

## Background

Dementia is a group of related symptoms caused by various diseases including Alzheimer’s disease (AD), vascular dementia (VD), and Lewy body dementia. These diseases impair people’s ability of memory, problem-solving and language, and interfere with their daily life [1]. A well-known risk factor for dementia is the increasing age, and most cases are those older adults aged over 65 [2]. According to the World Alzheimer Report 2018, there will be more than 152 million people suffering from dementia by the year 2050, and the total estimated worldwide cost of dementia will rise to 2 trillion by the year 2030 [3]. An inevitable aging society will bring great challenges to healthcare systems globally. At present, there is no cure for dementia, and medications are used to manage symptoms [4]. Thus, slowing the progress of dementia and maintaining patients’ cognition levels are of great importance [5].

Though the mechanism of dementia remains unclear, several studies show the relationship between oral health conditions and the progression of dementia. Specifically, periodontitis, a common oral disease, contributes to the progression of dementia [6–8]. A recently published review presented evidence that periodontal disease is associated with cognitive disorders (relative risk of 1.25) and cognitive impairment (relative risk of 3.01); and dementia (relative risk of 1.22) [9]. Periodontal pathogens and cytokines can induce neuroinflammation, a common pathological feature of dementia [6, 8, 10, 11]. Besides, tooth loss and poor mastication may contribute to dementia via decreasing prefrontal activations and cerebral blood flow [12, 13]. Dementia also has impacts on oral health. The amyloid-β, a major pathological molecular of dementia, disturbs the balance of oral microbiome [14]. Besides, people with dementia may have difficulties in taking routine oral hygiene practice such as toothbrushing and cleaning. This may worsen the oral health condition of people with dementia [15, 16]. Poor oral health and dementia may have interactive adverse impacts on each other, leading to a vicious circle. However, the rationale behind this is worth further investigation. It is necessary to find out the role of oral health interventions in maintaining people’s cognition levels and delaying the progression of dementia.

Aiming to improve people’s cognition levels, researchers have made efforts to provide a wide range of oral health interventions. However, current research findings are inconclusive. A clinical trial conducted in Japan showed that various oral health interventions such as oral hygiene instructions and oral function exercises were effective in improving oral health and executive function of cognitive function assessed via Trail Making Test (TMT), but this improvement could not be confirmed with another measurement scale, Mini-Mental State Examination (MMSE) [17]. Some studies found that daily oral care, provision of denture prosthesis and periodontal treatment could slow down the progress of cognition impairment, reduce the risk of developing dementia and improve patients’ cognition [18–20]. Despite this, some studies found that masticatory muscle training could not slow down the progress of cognition impairment [21, 22]. There are research gaps, e.g., does oral health intervention have a positive influence on the cognition of people with dementia? If so, what is the effective intervention? Who is supposed to provide the intervention? To answer the questions, we conducted a systemic review to assess the evidence, and to compare the cognition status of people with dementia after they had received oral health interventions.

## Methods

### The PICO question of this study was defined as follows

For people with dementia (P), what is the effect of oral health interventions (I) on the change of their cognition levels (O) compared with those without oral health interventions received (C). As oral health interventions varied among different studies, we intended to provide a full-view picture for readers by including all possible oral health interventions such as oral hygiene care and oral exercises. This systematic review and meta-analysis was reported according to the standards of the Preferred Reporting Items for Systematic Review and Meta-Analysis (PRISMA) Statement [23].

### Data searching and extraction

Literature search was conducted in the databases PubMed, Web of Science, Embase, Cochrane library, and Dentistry and Oral Sciences by two independent reviewers from inception to 6 March 2024. Clinical studies reporting on the effect of oral health interventions on the cognition of people with dementia or cognitive impairment were identified. The searching strategy was (“oral health” OR “dental health” OR “oral intervention” OR “dental intervention” OR “oral treatment” OR “dental treatment” OR “periodontal treatment”) AND (“dementia” OR “cognitive impairment” OR “cognition decline” OR “cognition” OR “cognitive defect” OR “Alzheimer’s disease”) (Appendix 1). No gray literature was included in the present review. Studies and publications were excluded if they were (1) clinical study without oral health intervention provided; (2) clinical study not reporting the change of cognition in any data format; (3) clinical study not conducted in dementia or cognitive impairment population; (4) laboratory study; (5) review, conference abstract, comment, case report and protocol; and (6) not written in English.

After the removal of duplicates, two independent reviewers screened titles and abstracts of the identified references, and the potential eligible references were retrieved for full-text reading. The above same reviewers conducted data extraction of the included studies. The main data extracted were the mean and standard deviation (SD) values of the cognition index MMSE, and the sample size of each included study. The MMSE score is commonly used to measure cognition and to assess the degree of dementia. A score ranging from 21 to 25 is considered as mild dementia, 11 to 20 as moderate dementia, and 10 and below as severe dementia [24]. A lower MMSE score indicates a worse cognition level. Besides, related information, i.e., authors, study region, publication year, participant’s age, and oral health interventions in study and control groups, were extracted and summarized. If there were disagreements on study inclusion or data extraction, a third independent investigator would join the discussion to arrive at a consensus.

### Quality assessment

The Cochrane risk-of-bias tool for randomized trials (RoB 2), which considers five domains of potential bias, i.e., randomization process, deviations from the intended interventions, missing outcome data, outcome measurement, and selective reporting, was adopted in this review to assess the risk of bias of the included studies [25]. The overall risk of bias of an included study was considered as ‘low’ when all the five domains were assessed as low risk. The overall risk of bias was ‘high’ if at least one domain was at high risk. Apart from the above-mentioned conditions, the overall risk of bias of the study was considered with “some concerns”.

### Statistical analysis

The mean deviation (MD) was used to assess the change in cognition at follow-up. The MD was generated by subtracting the baseline MMSE score from the MMSE score at follow-up. Meta-analysis was conducted using the software Review Manager 5.2. The statistical heterogeneity among studies was assessed by I^2^ test and Chi^2^ test. I^2^ value more than 40% and Chi^2^ value less than 0.1 suggested a statistical heterogeneity according to the Cochrane guidance [25]. The random-effect model was used to combine the data of the included studies. The weighted mean deviation (WMD) with 95% confidence interval (CI) was used to study the effect of oral health interventions on the cognition of these included studies. The inverse variance method was used to calculate the WMD. The statistical significance level for all tests was set at 5%.

## Results

### Study selection

**Fig. 1:**
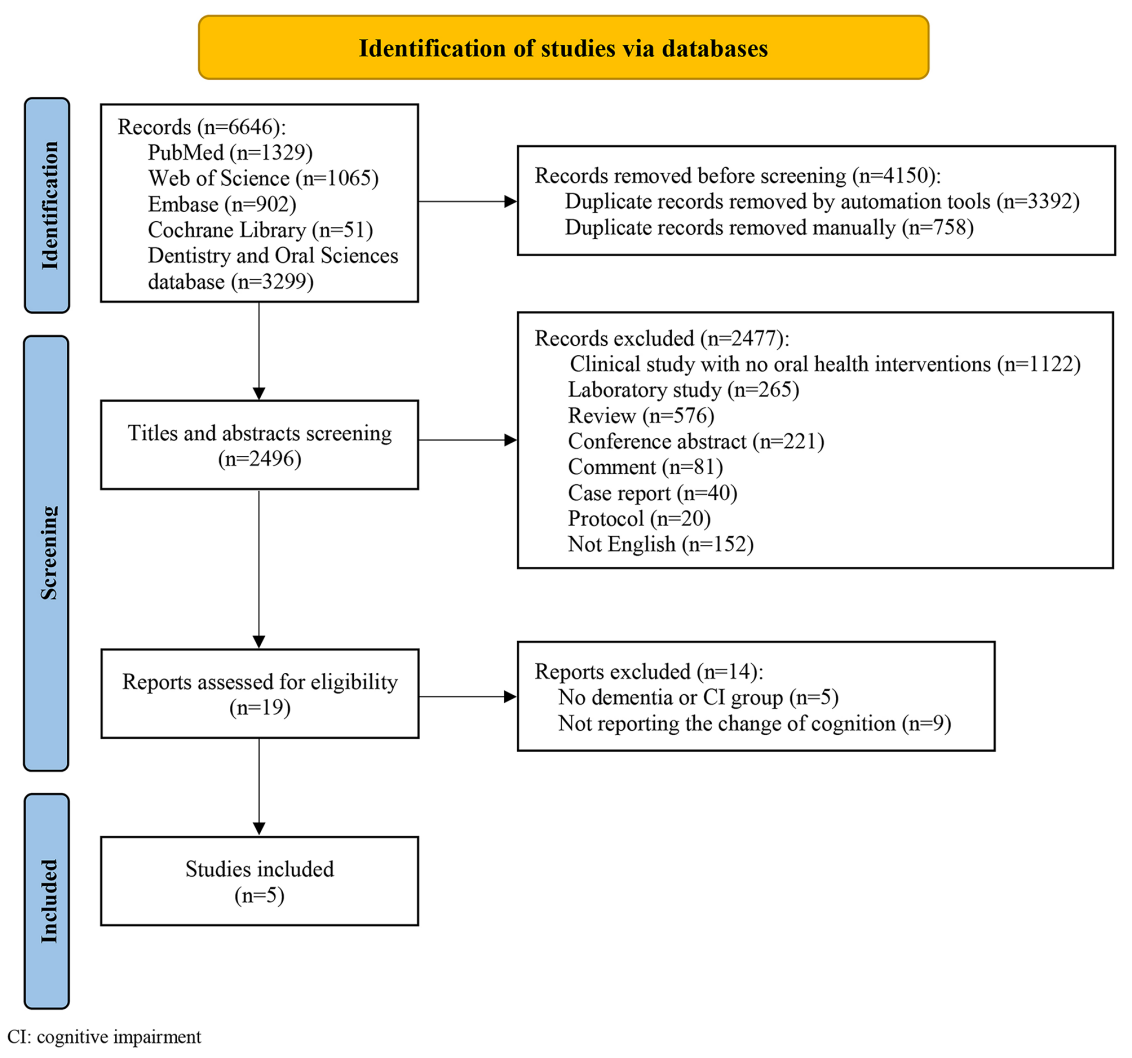
Flowchart of the selection process.CI: cognitive impairment

A total of 6646 references were identified by literature search (Fig. 1). After the removal of duplicates (*n* = 4150), titles and abstracts of the remaining references were screened. Subsequently, 2477 records were excluded for reasons, clinical study without oral health interventions provided (*n* = 1122), laboratory study (*n* = 265), review paper (*n* = 576), conference abstract (*n* = 221), comment (*n* = 81), case report (*n* = 40), protocol (*n* = 20), not written in English (*n* = 152). After full-text reading, 14 papers were further excluded because (1) not reporting the change in cognition (*n* = 9), and (2) study population without dementia or cognitive impairment (*n* = 5). Lastly, 5 studies were included in the present review [21, 26–29].

### Risk assessment of the included studies

**Fig. 2:**
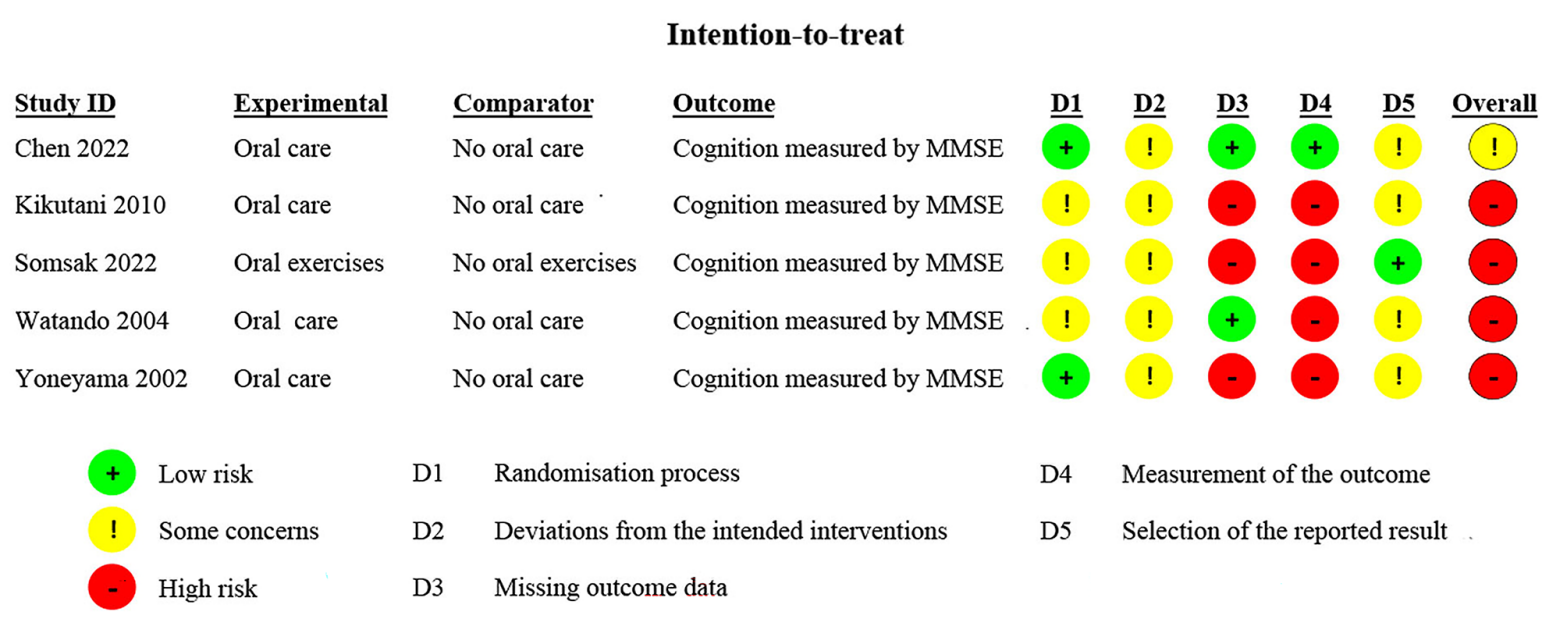
Assessment of risk of bias of the included studies. MMSE, Mini-Mental State Examination

Four of the included studies were ranked as high risk overall [21, 27–29], and the other one was assessed as “some concerns” [26] ( Fig. 2 and Appendix 2).

### Study characteristics

Table 1 shows the characteristics of the five included studies. Three studies were conducted in Japan [27–29], one was in China [26], and the other one was in Thailand [21]. The mean age of the participants ranged from 74 to 86. The follow-up period of the included studies ranged from 1 to 24 months [21, 26–29]. Four studies employed various oral hygiene practice as intervention, while the other study adopted oral exercises as intervention. Daily oral care was adopted in the control group of all five studies.

**Table 1:** Characteristics of the included studies

First author-Year	Location	Age (mean)	Sample size	Follow-up period	Intervention
Somsak-2022 [21]	Thailand	74.4	Baseline: Int: 11; Con: 11 Follow-up: Int: 10; Con: 9	3 months	Oral exercises
Chen-2022 [26]	China	82.9	Baseline: Int: 33; Con:33 Follow-up: Int: 33; Con:33	6 months	Oral hygiene practice
Kikutani-2010 [27]	Japan	82.0	Baseline: Int: 114; Con: 126 Follow-up: Int: 90; Con: 99	12 months	Oral hygiene practice
Watando-2004 [28]	Japan	86.1	Baseline: Int: 30; Con: 29 Follow-up: Int: 30; Con: 29	1 month	Oral hygiene practice
Yoneyama-2002 [29]	Japan	82.1	Baseline: Int: 184; Con: 182 Follow-up: Int: 170; Con: 152	24 months	Oral hygiene practice

Int: intervention group; Con: control group

Although oral hygiene practice was adopted as the oral health intervention in four studies, they used various ways to implement the practice in regards to toothbrushing method (frequency, provider, using toothpaste or not), agent for tongue, palatal and mucosa cleaning, and denture cleaning (Table 2). One study adopted oral exercises, i.e., tongue-strengthening, oral diadochokinesis and mouth-opening exercise as the intervention, where individuals were advised to take these exercises three days per week (on non-consecutive days) for 3 months [21].

**Table 2:** Details of the oral hygiene practice of the four included studies

	Chen-2022 [26]	Kikutani-2010 [27]	Watando-2004 [28]	Yoneyama-2002 [29]
**Toothbrushing**	With toothpaste	Without toothpaste	Without toothpaste	Without toothpaste
**Tongue**,** palate and mucosa cleaning**	Using sterile cotton swabs moistened with 0.2% chlorhexidine gluconate	Using toothbrush only	Using toothbrush only	Using an applicator with a minimum amount of povidone iodine (1%)
**Denture cleaning**	Unclear	Cleaned with a denture brush every day and with denture cleanser once a week	Cleaned with a denture brush every day and with denture cleanser once a week	Cleaned with a denture brush every day and with denture cleanser once a week
**Cleaning times**	Once a day, after dinner	After each meal	After each meal	After each meal
**Oral cleaning providers**	Participants themselves	Nurses or caregivers	Nurses or caregivers	Nurses or caregivers
**Professional oral care**	Unclear	Yes, once a week	Yes, once a week	Yes, once a week

Professional oral care referred to dentists or dental hygienists administering professional care such as plaque and calculus control as necessary once a week for the intervention group

### Cognition of participants in the included studies

At baseline, participants’ cognition status varied among different studies. The mean MMSE score in the intervention group ranged from 12.80 ± 9.31 to 20.00 ± 1.39 (Table 3). At follow-up, two studies [21, 28] presented increased MMSE scores in the intervention group, with positive MD values (MD = 0.60, MD = 0.90, respectively). However, one study [21] failed to show significant differences between intervention and control groups (intervention vs. control, 0.60 vs. 0.40, *p* = 0.895). Another three studies [26, 27, 29] reported decreased MMSE scores in both intervention and control groups with negative MD values, while intervention groups had lower absolute MD values compared with control groups (intervention vs. control, -0.18 vs. -0.75 (*p* < 0.05) [26], and − 1.50 vs. -3.00 (*p* < 0.05) [29], respectively), indicating less cognition deterioration in the intervention group. It should be pointed out that the study [27] only presented a diagram to show the changes in MMSE scores at 6-month and 12-month follow-ups (i.e., the MMSE scores decreased), but did not report the exact values of the MMSE scores at follow-ups.

**Table 3:** The changes of MMSE scores (mean ± standard deviation) of the included studies

	Intervention group			Control group			*p* value
	Baseline	Follow-up	MD	Baseline	Follow-up	MD	
Somsak-2022 [21]	15.60 ± 3.20	**16.20 ± 4.20**	0.60	15.70 ± 3.30	**16.10 ± 4.20**	0.40	0.895
Chen-2022 [26]	20.00 ± 1.39	**19.82 ± 1.57**	-0.18	19.36 ± 1.57	**18.61 ± 1.58**	-0.75	0.003
Kikutani-2010 [27]	18.20 ± 5.10	**NR**	**/**	17.80 ± 5.70	**NR**	**/**	< 0.05
Watando-2004 [28]	12.80 ± 9.31	**13.70 ± 9.86**	0.90	13.80 ± 10.23	**13.60 ± 9.69**	-0.20	> 0.05
Yoneyama-2002 [29]	14.30 ± 6.90	**12.80 ± 4.90**	-1.50	15.00 ± 8.40	**12.00 ± 5.90**	-3.00	< 0.05

MMSE: mini-mental status exam; MD, mean deviation; NR, exact values were not reported

*p* values were derived from the original paper, and they indicated the comparison of the MMSE scores between the intervention group and the control group at the follow-up

### Meta-analysis

**Fig. 3:**
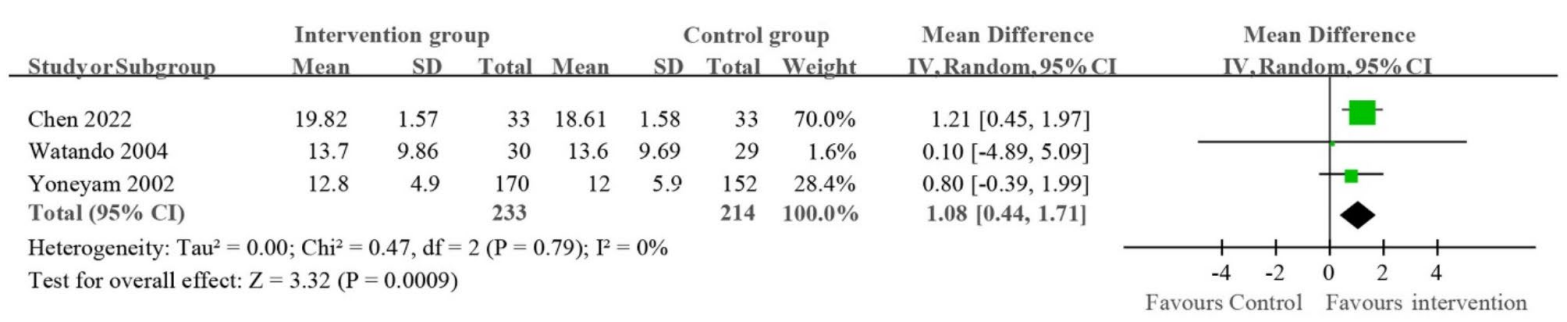
Forest plot of cognition difference between intervention and control groups

Data of the three studies [26, 28, 29] reporting on the change in MMSE scores after receiving oral hygiene care were extracted for further meta-analysis. It should be pointed out that we excluded the data of the study [21] from meta-analysis because the oral health intervention adopted in the study [21] was oral exercises. Due to the different nature of the two interventions, i.e., oral exercises and oral hygiene care, it is not appropriate to pool the data to conduct meta-analysis. Figure 3 shows the forest plot of the cognition difference between the intervention and control groups. The mean difference (95% CI) of MMSE scores between the intervention and control groups ranged from 0.10 (-4.89, 5.09) to 1.21 (0.45, 1.97), and the WMD was 1.08 (0.44, 1.71) favoring intervention.

## Discussion

In the present review, the findings of the meta-analysis show that the cognition status of intervention group is better than that of control group, favoring intervention (oral hygiene care). With limited evidence, we find that the provision of oral hygiene care as the oral health intervention has a beneficial effect on the cognition of people with dementia as assessed by MMSE scores. One study reported increased MMSE score (improved cognition level) in the intervention group at follow-up [28]. As for other included studies, even though reduced MMSE scores (worse cognition level) in both intervention and control groups can be observed at follow-ups, the cognition impairment is less severe in the intervention groups, which implies the potential benefits of provision of oral hygiene care [26, 29]. It should be pointed out that three studies were eligible to be included in the meta-analysis, but two [28, 29] out of the three studies were assessed with high risk of bias. Despite this, all the three studies showed the same direct, i.e., favoring oral hygiene care as the intervention. Even though we excluded the two high risk studies, this would not change the direct of the conclusion, still in favor of the intervention. The study [27] cannot be included in meta-analysis because it only presented a diagram to show the changes in MMSE scores at 6-month and 12-month follow-ups, but no exact values of the MMSE scores at follow-ups were reported. Thus, no data could be extracted for meta-analysis. Indeed, we did not exclude the study from the present review because we would like to provide readers a comprehensive summary of the current available evidence. On the other hand, current evidence fails to show positive effect of oral exercises on cognition level of people with dementia. Home-based oral exercises are found to be effective for improving oral function in terms of tongue strength and tongue-lip motor function in people with mild to moderate dementia, but this could not help with their cognition status [21].

Although the rationale of the correlation between oral hygiene practice and maintenance of cognition level remains unclear, the promising finding is supported by related studies. In animal studies, oral infection could induce cognitive impairment in mice by increasing the neuroinflammation and amyloid-β accumulation [30, 31], while inhibiting oral infection could alleviate cognitive impairment by decreasing the neuroinflammation and amyloid-β accumulation [32, 33]. Besides, periodontitis-related salivary microbiota aggravated AD pathogenesis through crosstalk of the gut-brain axis in APP^swe^/PS1^ΔE9^ (PAP) transgenic mice [34]. Meanwhile, *Lactobacillus pentosus* and *Bifidobacterium bifidum*, probiotics to treat periodontitis, suppressed cognitive impairment behaviors in the *Porphyromonas gingivalis* induced cognitive impairment mice model via regulating gut microbiota [35]. Moreover, tooth loss could induce a reduction of pyramidal cells in brain areas related to memory, learning and cognition in mice and a volume reduction in the hippocampus in human brains [36, 37]. Dentures for rats, on the contrary, could significantly increase the pyramidal cell density in hippocampal subfields, and improve the spatial learning and memory in rats [38]. Further, poor chewing could activate the hypothalamic-pituitary-adrenal (HPA) axis, leading to a hippocampal neurogenesis hyperactivity and eventually inducing a cognitive impairment [39]. While masticatory stimulation could attenuate the hyperactivity of the HPA axis, and alleviate cognitive deficits [39]. Thus, the findings of animal studies showed possible improvement and/or maintenance of cognition levels through different oral health interventions.

Despite this, it is found that the effects of oral health interventions on cognition were reported inconsistently among different clinical studies. This may be explained by three reasons. First, the type of dementia was different in the included studies. One study selected AD patients [26], while another study recruited AD patients, VD patients and mixed dementia patients [21]. The ability of AD patients to carry out simple tasks declines slowly as time goes by, while the ability declines sharply in VD patients [40, 41]. Besides, VD patients have a common concomitant symptom, physical disability, such as weakness or paralysis on one side of the body [42]. Different dementia types and characteristics may influence participants’ cooperation with oral health interventions, thus, differences in cognition changes may be detected among different studies. Second, the severity of dementia was different among the included studies. Three studies selected dementia individuals varying from mild to severe dementia [27–29], while one study only included individuals with mild dementia [26]. The different severity of dementia may result in a difference in the completion of oral health intervention and further lead to different outcomes perceived. Specifically, moderate and severe dementia patients could refuse to receive oral health interventions, and/or show limited cooperation, while patients with mild dementia had better cooperation [43, 44]. Third, the follow-up period of the included studies varied, ranging from 1 to 24 months. It is concerned that a short period may not be sufficient to observe the effect of the intervention. As we observed, three studies using the same oral health interventions with different follow-up periods showed inconsistent results [27–29]. The study with 1 month follow-up period reported no significant change [28], while the other two studies with longer follow-up periods found significant changes in cognition after provision of interventions [27, 29].

Although the present study was conducted based on up-to-date evidence, limitations of the included clinical studies should be pointed out, and the findings should be interpreted with caution. First, oral health status before and after intervention was not assessed and reported by the included studies. Oral health interventions are expected to improve oral health condition and function, so as to further improve and/or maintain cognition of dementia patients. Without assessment of oral health status, we have no clue regarding the effectiveness of oral health interventions, and further we couldn’t relate cognition changes with oral health interventions. Second, it is not clear whether the included studies have controlled confounders, such as depression and physical disability. Depression leads to a loss of motivation, and this will further affect the individual’s compliance with the intervention [45]. Moreover, physical disability compromises the patient’s ability to conduct daily oral hygiene practice, which would further influence the effect of oral hygiene interventions on cognition [46]. Polypharmacy (5 or more drugs) and malnutrition is another issue worth noting in dementia population. Several medications may negatively affect nutritional status via different mechanisms [47], and polypharmacy was found significantly associated with the incidence of dementia [48]. Thus, it is recommended to consider the above-mentioned factors in future studies. Third, although the MMSE score is commonly used in studies to measure cognition level, its sensitivity to detect small changes in cognition has been questioned [49]. It is suggested that cognition assessment tools with high sensitivity should be used to monitor the changes in cognition in future interventional studies. For example, the Montreal Cognitive Assessment (MoCA) may be a good tool to measure cognitive function [50]. Besides, various indirect changes, including brain activities, cerebral blood flow, and pathological features, could also be used to assist in the assessment of cognition changes. Overall, more well-designed high-quality clinical trials are needed to investigate the effect of oral health interventions on the cognition of people with dementia. Future studies should take several factors into consideration, for instance, causes and severity of dementia, oral health conditions (e.g., functional natural dentition vs. edentulous jaw), polypharmacy, and nutritional status. Strong and reliable evidence is demanded to guide clinicians as well as patients to take effective measures to slow down the cognition decline.

## Conclusion

With limited evidence, oral hygiene care may play a positive role in maintaining the cognition level of people with dementia. However, further studies are needed to provide direct evidence on the effectiveness of oral health interventions on oral health conditions as well as cognition status, and to disclose the rationale behind it.

### Electronic supplementary material

Below is the link to the electronic supplementary material.

Supplementary Material 1

Supplementary Material 2

## Data Availability

Data is provided within the manuscript and supplementary information files.

## Abbreviations

AD: Alzheimer’s disease
CI: Confidence interval
HPA: Hypothalamic-pituitary-adrenal axis
MD: Mean deviation
MMSE: Mini-Mental State Examination
MoCA: Montreal Cognitive Assessment
SD: Standard deviation
VD: Vascular dementia
WMD: Weighted mean difference
