# Supplementary material for: Effects of oral health interventions on cognition of people with dementia: a systematic review with meta-analysis
Source: BMC Oral Health. 2024 Sep 3;24:1030. doi: 10.1186/s12903-024-04750-4 (PMC11370033; doi:10.1186/s12903-024-04750-4)
Supplement: Supplementary file 1 — Supplementary Material 1 [file 12903_2024_4750_MOESM1_ESM.docx]

**Appendix-1. The searching strategy of different databases**

| **Pubmed** | |
| --- | --- |
| #1 | "oral health" OR "dental health" OR "oral intervention" OR "dental intervention" OR "oral treatment" OR "dental treatment" OR "periodontal treatment" |
| #2 | "dementia" OR "cognitive impairment" OR "cognition decline" OR "cognition" OR "cognitive defect" OR "Alzheimer's disease" |
| #1 AND #2 | 1329 |
| **Web of science** | |
| #1 | TS=("oral health" OR "dental health" OR "oral intervention" OR "dental intervention" OR "oral treatment" OR "dental treatment" OR "periodontal treatment") |
| #2 | TS=("dementia" OR "cognitive impairment" OR "cognition decline" OR "cognition" OR "cognitive defect" OR "Alzheimer's disease") |
| #1 AND #2 | 1065 |
| **Embase** | |
| #1 | ‘oral health’ ti,ab,kw OR ‘dental health’ ti,ab,kw OR ‘oral intervention’ ti,ab,kw OR ‘dental intervention’ ti,ab,kw OR ‘oral treatment’ ti,ab,kw OR ‘dental treatment’ ti,ab,kw OR ‘periodontal treatment’ ti,ab,kw |
| #2 | ‘dementia’ ti,ab,kw OR ‘cognitive impairment’ ti,ab,kw OR ‘cognition decline’ ti,ab,kw OR ‘cognition’ ti,ab,kw OR ‘cognitive defect’ ti,ab,kw OR ‘Alzheimer's disease’ ti,ab,kw |
| #1 AND #2 | 902 |
| **Cochrane reviews** | |
| #1 | ‘oral health’ OR ‘dental health’ OR ‘oral intervention’ OR ‘dental intervention’ OR ‘oral treatment’ OR ‘dental treatment’ OR ‘periodontal treatment’ in Title Abstract Keyword |
| #2 | ‘dementia’ OR ‘cognitive impairment’ OR ‘cognition decline’ OR ‘cognition’ OR ‘cognitive defect’ OR ‘Alzheimer's disease’ in Title Abstract Keyword |
| #1 AND #2 | 51 |
| **Dentistry and Oral Sciences** | |
| #1 | "oral health" OR "dental health" OR "oral intervention" OR "dental intervention" OR "oral treatment" OR "dental treatment" OR "periodontal treatment" |
| #2 | "dementia" OR "cognitive impairment" OR "cognition decline" OR "cognition" OR "cognitive defect" OR "Alzheimer's disease" |
| #1 AND #2 | 3299 |
