# Supplementary material for: Effects of oral health interventions on cognition of people with dementia: a systematic review with meta-analysis
Source: BMC Oral Health. 2024 Sep 3;24:1030. doi: 10.1186/s12903-024-04750-4 (PMC11370033; doi:10.1186/s12903-024-04750-4)
Supplement: Supplementary file 2 — Supplementary Material 2 [file 12903_2024_4750_MOESM2_ESM.pdf]

**Unique ID**	1	**Study ID**	Chen 2022	**Assessor**	ghy
**Ref or Label**	Effects of oral health intervention strategies on cognition and microbiota alterations in pa	**Aim**	assignment to intervention (the 'intention-to-treat' effect)		
**Experimental**	Oral care	**Comparator**	No oral care	**Source**	Journal article(s)
**Outcome**	Cognition measured by MMSE	**Results**	MMSE	**Weight**	
**Domain**	**Signalling question**			**Response**	**Comments**
**Bias arising from the randomization process**	1.1 Was the allocation sequence random?			Y	A random sequence was generated using a table of random numbers. Each of the random numbers was assigned using non-transparent sealed envelopes.
	1.2 Was the allocation sequence concealed until participants were enrolled and assigned to interventions?			Y	
	1.3 Did baseline differences between intervention groups suggest a problem with the randomization process?			N	No statistically significant differences were found between the intervention and control groups for all characteristics.
	**Risk of bias judgement**			**Low**	A random sequence was generated using a table of random numbers.Each of the random numbers was assigned using non-transparent sealed envelopes.No statistically significant differences were found between the intervention and control groups for all characteristics
**Bias due to deviations from intended interventions**	2.1.Were participants aware of their assigned intervention during the trial?			PY	Oral care might not be blind in this trail.
	2.2.Were carers and people delivering the interventions aware of participants' assigned intervention during the trial?			PY	
	2.3. If Y/PY/NI to 2.1 or 2.2: Were there deviations from the intended intervention that arose because of the experimental context?			PY	Participants might be unhappy becasue of the different interventions.
	2.4 If Y/PY to 2.3: Were these deviations likely to have affected the outcome?			PY	Participants might have different attitudes to the interventions.
	2.5. If Y/PY/NI to 2.4: Were these deviations from intended intervention balanced between groups?			PY	Participants might have different attitudes to the interventions.
	2.6 Was an appropriate analysis used to estimate the effect of assignment to intervention?			Y	Intention to treatment analyse
	2.7 If N/PN/NI to 2.6: Was there potential for a substantial impact (on the result) of the failure to analyse participants in the group to which they were randomized?			NA	
	**Risk of bias judgement**			**Some concerns**	
**Bias due to missing outcome data**	3.1 Were data for this outcome available for all, or nearly all, participants randomized?			Y	There is no missing data.
	3.2 If N/PN/NI to 3.1: Is there evidence that result was not biased by missing outcome data?			NA	
	3.3 If N/PN to 3.2: Could missingness in the outcome depend on its true value?			NA	
	3.4 If Y/PY/NI to 3.3: Is it likely that missingness in the outcome depended on its true value?			NA	
	**Risk of bias judgement**			**Low**	
**Bias in measurement of the outcome**	4.1 Was the method of measuring the outcome inappropriate?			N	Mini-Mental State Examination (MMSE) was used to assess the changes of the cognition.
	4.2 Could measurement or ascertainment of the outcome have differed between intervention groups?			N	Mini-Mental State Examination (MMSE) was used to assess the changes of the cognition.
	4.3 Were outcome assessors aware of the intervention received by study participants?			N	The data collector, sample collector, and data analyst were blinded to the study groups to avoid the subjective factors of the researcher from interfering with the test results.
	4.4 If Y/PY/NI to 4.3: Could assessment of the outcome have been influenced by knowledge of intervention received?			NA	
	4.5 If Y/PY/NI to 4.4: Is it likely that assessment of the outcome was influenced by knowledge of intervention received?			NA	
	**Risk of bias judgement**			**Low**	
**Bias in selection of the reported result**	5.1 Were the data that produced this result analysed in accordance with a pre-specified analysis plan that was finalized before unblinded outcome data were available for analysis?			PN	Measurement data were tested by the Shapiro-Wilk test. For measurement data, no extreme skewness or extreme outliers were observed, and therefore the data could be described by mean& standard deviation. No information.
	5.2 ... multiple eligible outcome measurements (e.g. scales, definitions, time points) within the outcome domain?			PN	Possible no, and only MMSE was reported.
	5.3 ... multiple eligible analyses of the data?			PN	Possible no.
	**Risk of bias judgement**			**Some concerns**	
**Overall bias**	**Risk of bias judgement**			**Some concerns**	

**Unique ID**	2	**Study ID**	Somsak 2022	**Assessor**	ghy
**Ref or Label**	Oral exercises significantly improve oral functions in people with mild and moderate [illegible]	**Aim**	assignment to intervention (the 'intention-to-treat' effect)		
**Experimental**	Oral exercises	**Comparator**	No oral exercises	**Source**	Journal article(s); Trial protocol
**Outcome**	Cognition measured by MMSE	**Results**	MMSE	**Weight**	
**Domain**	**Signalling question**			**Response**	**Comments**
**Bias arising from the randomization process**	1.1 Was the allocation sequence random?			N	Odd-numbered participants were in the experimental group (n = 11), while even numbered participants were in the control group (n = 11).
	1.2 Was the allocation sequence concealed until participants were enrolled and assigned to interventions?			N	
	1.3 Did baseline differences between intervention groups suggest a problem with the randomization process?			N	The data of participants in both experimental and control groups were not significantly different.
	**Risk of bias judgement**			**Some concerns**	
**Bias due to deviations from intended interventions**	2.1.Were participants aware of their assigned intervention during the trial?			Y	This study was an unblind clinical trial.
	2.2.Were carers and people delivering the interventions aware of participants' assigned intervention during the trial?			Y	
	2.3. If Y/PY/NI to 2.1 or 2.2: Were there deviations from the intended intervention that arose because of the experimental context?			PY	Different intervention might have an effect on the emotion.
	2.4 If Y/PY to 2.3: Were these deviations likely to have affected the outcome?			PY	The emotion might affect the cognition.
	2.5. If Y/PY/NI to 2.4: Were these deviations from intended intervention balanced between groups?			PY	Participants might have different concerns on interventions.
	2.6 Was an appropriate analysis used to estimate the effect of assignment to intervention?			Y	Intention-to-treatment analysis
	2.7 If N/PN/NI to 2.6: Was there potential for a substantial impact (on the result) of the failure to analyse participants in the group to which they were randomized?			NA	
	**Risk of bias judgement**			**Some concerns**	

Bias due to missing outcome data	3.1 Were data for this outcome available for all, or nearly all, participants randomized?	PN	Three subjects (1 in the experimental group and 2 in the control group) did not complete all data due to the COVID-19 pandemic (1 in the experimental group and 1 in the control group) and behavioural problems (1 in the control group)
	3.2 If N/PN/NI to 3.1: Is there evidence that result was not biased by missing outcome data?	PN	There was no evidence that the missing outcome could not impact the effect
	3.3 If N/PN to 3.2: Could missingness in the outcome depend on its true value?	PY	Age was unclear in the missing data and vascular dementia in the two groups were also unclear.
	3.4 If Y/PY/NI to 3.3: Is it likely that missingness in the outcome depended on its true value?	PY	
	**Risk of bias judgement**	**High**	
Bias in measurement of the outcome	4.1 Was the method of measuring the outcome inappropriate?	N	Mini-Mental State Examination (MMSE) was used to assess the changes of the cognition.
	4.2 Could measurement or ascertainment of the outcome have differed between intervention groups?	N	Mini-Mental State Examination (MMSE) was used to assess the changes of the cognition.
	4.3 Were outcome assessors aware of the intervention received by study participants?	Y	This study was an unblind clinical trial.
	4.4 If Y/PY/NI to 4.3: Could assessment of the outcome have been influenced by knowledge of intervention received?	PY	As MMSE was the participant-reported outcome and involved some judgement, thus the assessment of the outcome influenced by knowledge of intervention received.
	4.5 If Y/PY/NI to 4.4: Is it likely that assessment of the outcome was influenced by knowledge of intervention received?	PY	
	**Risk of bias judgement**	**High**	
Bias in selection of the reported result	5.1 Were the data that produced this result analysed in accordance with a pre-specified analysis plan that was finalized before unblinded outcome data were available for analysis?	Y	The data were in accordance with a pre-specified analysis plan.
	5.2 ... multiple eligible outcome measurements (e.g. scales, definitions, time points) within the outcome domain?	N	Only the MMSE was reported.
	5.3 ... multiple eligible analyses of the data?	N	No.
	**Risk of bias judgement**	**Low**	
**Overall bias**	**Risk of bias judgement**	**High**	

**Unique ID**	3	**Study ID**	Watando 2004	**Assessor**	ghy
**Ref or Label**	Daily oral care and cough reflex sensitivity in elderly nursing home patients	**Aim**	assignment to intervention (the 'intention-to-treat' effect)		
**Experimental**	Oral care	**Comparator**	No oral care	**Source**	Journal article(s)
**Outcome**	Cognition measured by MMSE	**Results**	MMSE	**Weight**	

Domain	Signalling question	Response	Comments
Bias arising from the randomization process	1.1 Was the allocation sequence random?	Y	Sixty patients were randomly assigned to an intensive oral care (intervention) group or no intensive oral care (control) group in February 2003 using a random-numbers table, and were investigated for 1 month.
	1.2 Was the allocation sequence concealed until participants were enrolled and assigned to interventions?	NI	
	1.3 Did baseline differences between intervention groups suggest a problem with the randomization process?	NI	One patient from the control group was excluded from analysis because he died from stroke before the study finished and we didn't know the stauts of the patient.
	**Risk of bias judgement**	**Some concerns**	
Bias due to deviations from intended interventions	2.1.Were participants aware of their assigned intervention during the trial?	PY	Oral care couldn't be blind during the trail.
	2.2.Were carers and people delivering the interventions aware of participants' assigned intervention during the trial?	PY	
	2.3. If Y/PY/NI to 2.1 or 2.2: Were there deviations from the intended intervention that arose because of the experimental context?	PY	Different intervention might have an effect on the emotion.
	2.4 If Y/PY to 2.3: Were these deviations likely to have affected the outcome?	PY	The emotion might affect the cognition.
	2.5. If Y/PY/NI to 2.4: Were these deviations from intended intervention balanced between groups?	PY	Participants might have different concerns on interventions.
	2.6 Was an appropriate analysis used to estimate the effect of assignment to intervention?	Y	Modified intention-to-treatment analysis
	2.7 If N/PN/NI to 2.6: Was there potential for a substantial impact (on the result) of the failure to analyse participants in the group to which they were randomized?	NA	
	**Risk of bias judgement**	**Some concerns**	
Bias due to missing outcome data	3.1 Were data for this outcome available for all, or nearly all, participants randomized?	PY	Only 1 participants was excluded because he died from stroke before the study finished.
	3.2 If N/PN/NI to 3.1: Is there evidence that result was not biased by missing outcome data?	NA	
	3.3 If N/PN to 3.2: Could missingness in the outcome depend on its true value?	NA	
	3.4 If Y/PY/NI to 3.3: Is it likely that missingness in the outcome depended on its true value?	NA	
	**Risk of bias judgement**	**Low**	
Bias in measurement of the outcome	4.1 Was the method of measuring the outcome inappropriate?	N	Mini-Mental State Examination (MMSE) was used to assess the changes of the cognition.
	4.2 Could measurement or ascertainment of the outcome have differed between intervention groups?	N	Mini-Mental State Examination (MMSE) was used to assess the changes of the cognition.
	4.3 Were outcome assessors aware of the intervention received by study participants?	NI	There is no information about this.
	4.4 If Y/PY/NI to 4.3: Could assessment of the outcome have been influenced by knowledge of intervention received?	PY	As MMSE was the participant-reported outcome and involved some judgement, thus the assessment of the outcome influenced by knowledge of intervention received.
	4.5 If Y/PY/NI to 4.4: Is it likely that assessment of the outcome was influenced by knowledge of intervention received?	PY	
	**Risk of bias judgement**	**High**	
Bias in selection of the reported result	5.1 Were the data that produced this result analysed in accordance with a pre-specified analysis plan that was finalized before unblinded outcome data were available for analysis?	NI	There is no infromation about it.
	5.2 ... multiple eligible outcome measurements (e.g. scales, definitions, time points) within the outcome domain?	PN	Possible no, and only the MMSE was reported.
	5.3 ... multiple eligible analyses of the data?	PN	Possible no.
	**Risk of bias judgement**	**Some concerns**	
**Overall bias**	**Risk of bias judgement**	**High**	

**Unique ID**	4	**Study ID**	Yoneyama 2002	**Assessor**	ghy

**Ref or Label**		**Aim**	assignment to intervention (the 'intention-to-treat' effect)		
**Experimental**	Oral care	**Comparator**	No oral care	**Source**	Journal article(s)
**Outcome**	Cognition measured by MMSE	**Results**	MMSE	**Weight**	

**Domain**	**Signalling question**	**Response**	**Comments**
**Bias arising from the randomization process**	1.1 Was the allocation sequence random?	Y	The patients were randomly selected from the same floor and nursing team in each nursing home. Four hundred seventeen patients were randomly assigned to an oral care group or a no oral care group in September 1996 and were investigated for 2 years. Randomization was made from a random-numbers table, and the list was held independently of the investigators.
	1.2 Was the allocation sequence concealed until participants were enrolled and assigned to interventions?	PY	
	1.3 Did baseline differences between intervention groups suggest a problem with the randomization process?	NI	However, 51 patients were excluded from the analysis because they died from causes other than pneumonia during follow-up.
	**Risk of bias judgement**	**Low**	
**Bias due to deviations from intended interventions**	2.1.Were participants aware of their assigned intervention during the trial?	PY	The patients were randomly selected from the same floor and nursing team in each nursing home. Thus, the oral care might not be blind.
	2.2.Were carers and people delivering the interventions aware of participants' assigned intervention during the trial?	PY	
	2.3. If Y/PY/NI to 2.1 or 2.2: Were there deviations from the intended intervention that arose because of the experimental context?	PY	Different intervention might have an effect on the emotion.
	2.4 If Y/PY to 2.3: Were these deviations likely to have affected the outcome?	PY	The emotion might affect the cognition.
	2.5. If Y/PY/NI to 2.4: Were these deviations from intended intervention balanced between groups?	PY	Participants might have different concerns on interventions.
	2.6 Was an appropriate analysis used to estimate the effect of assignment to intervention?	Y	Modified intention-to-treatment analysis
	2.7 If N/PN/NI to 2.6: Was there potential for a substantial impact (on the result) of the failure to analyse participants in the group to which they were randomized?	NA	
	**Risk of bias judgement**	**Some concerns**	
**Bias due to missing outcome data**	3.1 Were data for this outcome available for all, or nearly all, participants randomized?	PN	Thirty people (16%) in the non–oral care group died and 14 (7%) in the oral care group died were excluded.
	3.2 If N/PN/NI to 3.1: Is there evidence that result was not biased by missing outcome data?	N	As we didn't know the details of these 51 participants. Might many of them were dementia patients and all in the intervention group, which might impact the effect of the intervention.
	3.3 If N/PN to 3.2: Could missingness in the outcome depend on its true value?	PY	Age and other confounding factors were unclear.
	3.4 If Y/PY/NI to 3.3: Is it likely that missingness in the outcome depended on its true value?	PY	
	**Risk of bias judgement**	**High**	
**Bias in measurement of the outcome**	4.1 Was the method of measuring the outcome inappropriate?	N	Mini-Mental State Examination (MMSE) was used to assess the changes of the cognition.
	4.2 Could measurement or ascertainment of the outcome have differed between intervention groups?	N	Mini-Mental State Examination (MMSE) was used to assess the changes of the cognition.
	4.3 Were outcome assessors aware of the intervention received by study participants?	NI	There was no information about this.
	4.4 If Y/PY/NI to 4.3: Could assessment of the outcome have been influenced by knowledge of intervention received?	PY	As MMSE was the participant-reported outcome and involved some judgement, thus the assessment of the outcome influenced by knowledge of intervention received.
	4.5 If Y/PY/NI to 4.4: Is it likely that assessment of the outcome was influenced by knowledge of intervention received?	PY	
	**Risk of bias judgement**	**High**	
**Bias in selection of the reported result**	5.1 Were the data that produced this result analysed in accordance with a pre-specified analysis plan that was finalized before unblinded outcome data were available for analysis?	NI	No information
	5.2 ... multiple eligible outcome measurements (e.g. scales, definitions, time points) within the outcome domain?	N	Possible no, and only MMSE was reported.
	5.3 ... multiple eligible analyses of the data?	N	Possible no.
	**Risk of bias judgement**	**Some concerns**	
**Overall bias**	**Risk of bias judgement**	**High**	

**Unique ID**	5	**Study ID**	Kikutani 2010	**Assessor**	ghy
**Ref or Label**		**Aim**	assignment to intervention (the 'intention-to-treat' effect)		
**Experimental**	Oral care	**Comparator**	No oral care	**Source**	
**Outcome**	Cognition measured by MMSE	**Results**	MMSE	**Weight**	

**Domain**	**Signalling question**	**Response**	**Comments**
**Bias arising from the randomization process**	1.1 Was the allocation sequence random?	Y	A total of 275 patients were randomly assigned to an oral care group or a no oral care group from September 2003 to August 2004.
	1.2 Was the allocation sequence concealed until participants were enrolled and assigned to interventions?	NI	
	1.3 Did baseline differences between intervention groups suggest a problem with the randomization process?	NI	No inforamtion about the baselin differences in the study.
	**Risk of bias judgement**	**Some concerns**	
**Bias due to deviations from intended interventions**	2.1.Were participants aware of their assigned intervention during the trial?	PY	Oral care couldn't be blind during the trail.
	2.2.Were carers and people delivering the interventions aware of participants' assigned intervention during the trial?	PY	
	2.3. If Y/PY/NI to 2.1 or 2.2: Were there deviations from the intended intervention that arose because of the experimental context?	PY	Different intervention might have an effect on the emotion.
	2.4 If Y/PY to 2.3: Were these deviations likely to have affected the outcome?	PY	The emotion might affect the cognition.
	2.5. If Y/PY/NI to 2.4: Were these deviations from intended intervention balanced between groups?	PY	Participants might have different concerns on interventions.
	2.6 Was an appropriate analysis used to estimate the effect of assignment to intervention?	Y	Modified intention-to-treatment analysis
	2.7 If N/PN/NI to 2.6: Was there potential for a substantial impact (on the result) of the failure to analyse participants in the group to which they were randomized?	NA	
	**Risk of bias judgement**	**Some concerns**	

**Bias due to missing outcome data**	3.1 Were data for this outcome available for all, or nearly all, participants randomized?	N	Twenty-four patients for the oral care group (n=114) and 27 patients for the no oral care group (n=126) were excluded from the analysis, because they were discharged from nursing homes, hospitalized, died or dropped out due to discontinuation of the examination.
	3.2 If N/PN/NI to 3.1: Is there evidence that result was not biased by missing outcome data?	N	As we didn't know the details of these missing participants.
	3.3 If N/PN to 3.2: Could missingness in the outcome depend on its true value?	PY	Possible yes.
	3.4 If Y/PY/NI to 3.3: Is it likely that missingness in the outcome depended on its true value?	PY	
	**Risk of bias judgement**	**High**	
**Bias in measurement of the outcome**	4.1 Was the method of measuring the outcome inappropriate?	N	Mini-Mental State Examination (MMSE) was used to assess the changes of the cognition.
	4.2 Could measurement or ascertainment of the outcome have differed between intervention groups?	N	Mini-Mental State Examination (MMSE) was used to assess the changes of the cognition.
	4.3 Were outcome assessors aware of the intervention received by study participants?	NI	There was no information about this.
	4.4 If Y/PY/NI to 4.3: Could assessment of the outcome have been influenced by knowledge of intervention received?	PY	As MMSE was the participant-reported outcome and the involved some judgement, thus the assessment of the outcome influenced by knowledge of intervention received.
	4.5 If Y/PY/NI to 4.4: Is it likely that assessment of the outcome was influenced by knowledge of intervention received?	PY	
	**Risk of bias judgement**	**High**	
**Bias in selection of the reported result**	5.1 Were the data that produced this result analysed in accordance with a pre-specified analysis plan that was finalized before unblinded outcome data were available for analysis?	NI	There is no infromation about it.
	5.2 ... multiple eligible outcome measurements (e.g. scales, definitions, time points) within the outcome domain?	PN	Possible no, and only MMSE was reported.
	5.3 ... multiple eligible analyses of the data?	PN	Possible no.
	**Risk of bias judgement**	**Some concerns**	
**Overall bias**	**Risk of bias judgement**	**Some concerns**	
